# Editorial: Heme physiology and pathology

**DOI:** 10.3389/fphys.2025.1731803

**Published:** 2025-11-07

**Authors:** Elena Di Pierro, Tiago L. Duarte

**Affiliations:** 1 SC Medicina ad Indirizzo Metabolico, Fondazione IRCCS Ca’ Granda Ospedale Maggiore Policlinico, Milan, Italy; 2 i3S–Instituto de Investigação e Inovação em Saúde, Universidade do Porto, Porto, Portugal

**Keywords:** heme, hemopexin, microbiota, inflammation, oxigen delivery

Heme is an essential prosthetic group for a variety of proteins involved in vital physiological functions in the body, such as oxygen transport, drug metabolism, biosynthesis of steroids, signal transduction, antioxidant defense and mitochondrial respiration. However, when unbound, free heme becomes a potent pro-oxidant and inflammatory danger molecule. Maintaining heme homeostasis, therefore, is crucial for tissue integrity and systemic health.

The liver plays a central role in heme metabolism by significantly contributing to heme synthesis, heme detoxification, and recycling of heme iron. In the comprehensive review “*Heme (Dys)homeostasis and Liver Disease*,” Duarte et al. outline how the delicate balance of heme production, utilization, and degradation influences hepatic health. They describe the regulation of ALAS1, the rate-limiting enzyme of hepatic heme synthesis, and discuss how disruptions in heme turnover—whether through porphyrias, hemolysis, or impaired catabolism—trigger oxidative stress, inflammation, and ferroptosis. The review integrates recent data linking heme excess to non-alcoholic fatty liver disease, acute liver failure, and hepatocellular carcinoma, emphasizing therapeutic opportunities in modulating heme metabolism and iron handling to restore redox equilibrium and prevent cell death.

Silveira et al. (“*Targeting Heme in Sickle Cell Disease: New Perspectives on Priapism Treatment*”) address the pathological role of free heme in the vascular complications of sickle cell disease (SCD), focusing on priapism. Chronic hemolysis in SCD releases free hemoglobin and heme, which drive endothelial activation through TLR4 and oxidative pathways, disrupting the nitric-oxide (NO)–cyclic guanosine monophosphate (cGMP) axis that regulates penile smooth muscle tone. The authors discuss therapeutic strategies aimed at neutralizing free heme or augmenting heme catabolism, such as hemopexin and haptoglobin supplementation, HO-1 induction, and modulation of the HO-CO-soluble guanylate cyclase (sGC) pathway. This perspective highlights heme toxicity as a treatable driver of SCD complications beyond vaso-occlusion, suggesting translational potential for targeted heme-scavenging or signaling-based interventions.

In their original study, Smith et al. (“*Tracking Hemopexin Intracellularly and Defining Hemopexin Protein Interactomes in Human Immune and Liver Cell Models*”) provide a mechanistic exploration of hemopexin (HPX) uptake and trafficking in immune and hepatic cells, expanding the molecular understanding of how cells manage heme. Using confocal microscopy and proteomic mapping, they demonstrate that HPX colocalizes with transferrin and transferrin receptor 1 (TfR1) in Rab5-positive early endosomes of HL-60 neutrophil-like cells, indicating a clathrin-mediated internalization process. In hepatocytes, HPX interacts with LRP1, TfR1, and TfR2, suggesting cell-specific receptor usage and distinct outcomes for heme-loaded versus apo-HPX.

The review by Chen et al., “*Role of Gut Microbiota in Thalassemia: A Review of Therapeutic Prospects*,” connects systemic iron and heme dysregulation to intestinal microbiota alterations in thalassemia. Chronic transfusions and ineffective erythropoiesis cause iron overload, leading to a dysbiotic microbiota characterized by reduced diversity and depletion of butyrate-producing bacteria. This microbial imbalance contributes to intestinal inflammation, increased permeability, and systemic immune activation. The authors suggest that microbiome-directed therapies—such as probiotics, prebiotics, dietary interventions, or fecal microbiota transplantation—could complement chelation therapy by reducing inflammation and restoring metabolic balance. Their synthesis highlights the inter-organ communication among the gut, liver, and bone marrow, positioning microbiota modulation as a novel approch in hemoglobinopathy management.

Heme’s physiological role as an oxygen carrier is revisited in Dalne et al. (“*Evolution of the Oxyhemoglobin Dissociation Curve in COVID-19 Related ARDS Patients*”). The study quantifies changes in hemoglobin-oxygen affinity (corrected P50) in patients with COVID-19 acute respiratory distress syndrome (ARDS) compared with non-COVID ARDS controls. At ICU admission, COVID-19 ARDS patients exhibited a left-shifted dissociation curve—indicatiing higher oxygen affinity—which remained largely stable over the first 3 days and was not associated with mortality. These data clarify controversies about altered oxygen binding in COVID-19 and suggest that, despite profound hypoxemia, intrinsic hemoglobin function remains preserved. The findings refine our understanding of oxygen transport physiology under severe inflammatory stress.

In the study “*Alteration in the Number, Morphology, Function, and Metabolism of Erythrocytes in High-Altitude Polycythemia*,” Yu et al. use a hypobaric hypoxia rat model to characterize the adaptive and maladaptive responses of erythrocytes under chronic hypoxia. They report increased erythropoiesis and reduced erythrocyte death, resulting in marked erythrocytosis. Morphological abnormalities (acanthocytes, vesiculated cells) and elevated osmotic fragility indicate membrane instability. Metabolically, erythrocytes upregulate the CD73, adenosine, S1P, and 2,3-BPG pathways, reducing hemoglobin oxygen affinity to facilitate tissue oxygen unloading. These findings reveal how red cells remodel their structure and metabolism to optimize oxygen delivery at altitude, while also predisposing to viscosity-related complications.

This Research Topic, *Heme Physiology and Pathology*, presents six contributions covering the spectrum from molecular mechanisms of heme handling to clinical and pathophysiological implications in different diseases, including liver injury, sickle cell disease, thalassemia, high-altitude polycythemia, and COVID-19-related ARDS. Collectively, these papers highlight the dynamic roles of heme and hemoglobin in health and disease, and point toward novel diagnostic and therapeutic strategies targeting heme metabolism and signaling. The editors hope that this Research Topic will stimulate continued exploration into the physiological and pathological aspects of heme, ultimately advancing therapeutic innovation in red cell and systemic disorders.

